# Increasing Hospitalizations and General Practice Prescriptions for Community-onset Staphylococcal Disease, England

**DOI:** 10.3201/eid1505.070153

**Published:** 2008-05

**Authors:** Andrew Hayward, Felicity Knott, Irene Petersen, David M. Livermore, Georgia Duckworth, Amir Islam, Anne M. Johnson

**Affiliations:** * University College London Centre for Infectious Disease Epidemiology, London, UK; † Health Protection Agency, London, UK

**Keywords:** epidemiology, staphylococcal infections, community, trends, abscess, cellulitis, impetigo, osteomyelitis, pneumonia, bacteremia, research

## Abstract

Rates of hospital-acquired staphylococcal infection increased throughout the 1990s; however, information is limited on trends in community-onset staphylococcal disease in the United Kingdom. We used Hospital Episode Statistics to describe trends in hospital admissions for community-onset staphylococcal disease and national general practice data to describe trends in community prescribing for staphylococcal disease. Hospital admission rates for staphyloccocal septicemia, staphylococcal pneumonia, staphylococcal scalded-skin syndrome, and impetigo increased >5-fold. Admission rates increased 3-fold for abscesses and cellulitis and 1.5-fold for bone and joint infections. In primary care settings during 1991–2006, floxacillin prescriptions increased 1.8-fold and fusidic acid prescriptions 2.5-fold. The increases were not matched by increases in admission rates for control conditions. We identified a previously undescribed but major increase in pathogenic community-onset staphylococcal disease over the past 15 years. These trends are of concern given the international emergence of invasive community-onset staphylococcal infections.

## Abstract 2

Infections requiring hospitalization and community prescriptions have increased dramatically since 1989.

Harmless colonization of the nasal membranes and skin with *Staphylococcus aureus* is common in the community (*1*), but the organism can also cause a variety of infections (*2*). *S. aureus* is the most common cause of skin and soft tissue infections, including wound infections, abscesses, furuncles, carbuncles (*3*), and impetigo (*4*) and is a major cause of cellulitis (*5*). It can also cause deep-seated infections such as osteomyelitis (*6*), endocarditis, pneumonia, and septicemia (*2*). Toxigenic strains can cause toxic shock, staphylococcal scalded-skin syndrome (SSSS), and staphylococcal food poisoning (*2*).

More than 70 different potential virulence factors have been identified in *S. aureus,* including adhesins, exoenzymes, and exotoxins (*7*). Exfoliative toxins are associated with impetigo and SSSS (*8*), and production of Panton-Valentine leukocidin (PVL) toxin has been associated with invasive strains that cause abscesses, bone and joint infection, and pneumonia (*9**,**10*). In the 1950s, PVL production was associated with particularly invasive infections caused by the penicillin-resistant phage-type 80/81 strain (*11**,**12*). More recently, in 2006, a review of data on isolates referred to the Health Protection Agency’s Staphylococcal Reference Unit identified 27 deaths in 27 months from infections with invasive PVL-positive *S. aureus*. These deaths were in previously healthy people with community-onset pneumonia, bacteremia, or severe skin and soft tissue infection (*13*). Most were caused by methicillin-sensitive *S. aureus* (MSSA) strains. In view of these data and the wider concern about emerging community-onset methicillin-resistant *S. aureus* (MRSA) (*14**–**16*), we sought to determine whether there had been a generalized increase in 1) community-onset staphylococcal disease severe enough to merit hospitalization and 2) general practice (GP) antimicrobial drug prescribing for skin and soft tissue infections putatively caused by staphylococci.

## Methods

We analyzed Hospital Episode Statistics (HES) to identify trends in admissions for community-onset disease that were likely to be caused by pathogenic staphylococci. HES has been operating nationally, recording all admissions to NHS hospitals, since 1989. Inpatient admissions are coded by professional coders who used the International Classification of Diseases (ICD) version 9 before 1995 and version 10 subsequently. The main reason for admission is recorded as the “primary diagnostic code.” HES data have been widely used to examine time trends in disease and variations in practice and to make international comparisons (www.hesonline.nhs.uk).

We developed ICD-9 and ICD-10 code lists for the following infections: staphylococcal septicemia, staphylococcal pneumonia, abscess, furuncle, carbuncle, cellulitis, impetigo, bone and joint infections, and SSSS (Table 1). These infections were chosen because *S. aureus* is the identified or most likely causative organism (or, in the case of cellulitis, 1 of the most important organisms). We then obtained HES data extracts on admissions to hospitals where the primary diagnosis was 1 of these codes. These data covered all NHS hospitals in England from the 1989–1990 financial year to the 2003–2004 financial year (April 1 to the following March 31). Abscess and cellulitis codes were not well discriminated in ICD-9; many codes both identified abscess and cellulitis and thus precluded separate analysis before 1995. In ICD-10, the codes for abscess and cellulitis are separated. There was no code for SSSS in ICD-9, precluding analysis of trends for this condition before 1995.

**Table 1:** Comparison of trends in hospital admissions, England, 1989–90 to 2003–04*

Type of infection and ICD codes	No. admissions (age-standardized admission rate per 100,000 population)			Standardized admission ratios (95% CI)
Acute community-onset infections likely to be caused by staphylococci (ICD-9: ICD-10 codes)	1989–90	2003–04		2003–04 vs. 1989–90
Abscess, carbuncle, and furuncle and/or cellulitis (6800–6811, 6819–6829: L02.0–L03.9)	23,884 (50.0)	74,447(148.8)		2.98 (2.96–3.00)
Bone and joint infection (7300–7309, 7110: M86.0– M86.6, M86.8, M86.9, M00.0–M00.2,M00.8,M00.9)	4,104 (8.9)	6,700 (13.4)		1.57 (1.53–1.60)
Staphylococcal septicemia (381: A41.0–A41.2)	249 (0.5)	1,681 (3.3)		6.39 (6.10–6.71)
Impetigo (684: L01.0,L01.1)	199 (0.4)	1,108 (2.5)		5.92 (5.58–6.28)
Staphylococcal pneumonia (4824: J15.2)	109 (0.2)	568 (1.2)		5.04 (4.64–5.47)
	1995–96	2003–04		2003–04 vs. 1995–96
Cellulitis (NA: L03.0–L03.9)	24,388 (50.3)	49,980 (98.9)		1.97 (1.95–1.98)
Abscess, carbuncle, and furuncle (NA: L02.0–L02.9)	12,675 (26.1)	24,467 (49.3)		1.89 (1.86–1.91)
Staphylococcal scalded-skin syndrome (NA: L00.X)	149 (0.3)	747 (1.6)		5.27 (4.91–5.63)
Acute community-onset control conditions (ICD-9: ICD-10 codes)	1989–90	2003–04		2003–04 vs. 1989–90
Forearm fracture (8130–8135:S520–S529)	30,272 (63.2)	54,089(106.9)		1.69 (1.01–1.68)
Acute appendicitis (5400–5409, K35.0–K35.9)	30,946 (64.6)	30,324 (61.5)		0.95 (0.94–0.96)
Ingrown toenail (7030:L60.0)	16,606 (34.7)	11,182 (22.7)		0.65 (0.64–0.67)
Other septicemias (380–384, 388,389: A400–A409, A414, A415, A418, A419	3,793 (7.9)	12,873 (24.9)		3.15 (3.10–3.20)
Gastroenteritis/diarrhea of presumed infectious origin (91: A09.X)	8,416 (17.6)	6,528 (14.3)		0.81 (0.79–0.83)
Cholecystitis (5750: K81.0)	3,171 (6.6)	4,264 (8.3)		1.25 (1.21–1.28)
Conjunctivitis (3720–3724:H10.0–H10.9)	639 (1.3)	193 (0.3)		0.33 (0.29–0.38)
Viral pneumonia (4800–4809:J12.0–J12.9)	585 (1.2)	501 (1.1)		0.88 (0.81–0.96)
Erysipelas (35:A46.X)	373 (0.8)	357 (0.7)		0.89 (0.80–0.99)

*Years are financial years, April 1 to March 31. CI, confidence interval; ICD, International Classification of Diseases; NA, not available.

We obtained annual population denominator data from the Office for National Statistics (www.statistics.gov.uk) and used these to calculate 1) age-specific annual rates of admission for the different infections and 2) age-specific rate ratios comparing rates in 2003–04 with those in 1989–90 or, where data before 1995 were not available because of coding changes (abscesses, cellulitis, and SSSS), comparing rates in 2003–04 with those in 1995–96. We calculated 95% confidence intervals (CIs) by the delta method. We calculated annual age-standardized admission rates by using indirect standardization; the population of England in the earliest year of the trend was used as the baseline population (1989–90 for most staphylococcal conditions but 1995–96 for abscess, cellulitis, and SSSS). Standardized admission ratios and 95% CIs comparing 2003–04 rates with those in the earliest year of the trend were calculated by using indirect standardization to the baseline population (*17*). Age standardization was used to account for the fact that the age structure of the population had changed over the study period and to allow valid comparison of rates in different years.

To verify that observed trends were not part of a generalized increase in hospital admissions, we used HES data to examine trends in the following control conditions: appendicitis, cholecystitis, conjunctivitis, fractured forearm, gastroenteritis/diarrhea of presumed infectious origin, ingrown toenail, and erysipelas. These conditions were chosen as examples of acute community-onset conditions that are not normally caused by staphylococci. We also examined trends in hospitalizations for septicemia in which staphylococci were not identified as the causative organism.

We analyzed data from the Prescription Prescribing Authority, which collects information on all prescriptions issued by general practitioners and dispensed by community pharmacists and dispensing general practitioners (PACT data). The information collected includes the name of the drug and the number of items dispensed (an item is defined as each preparation on the prescription). The data cannot be linked routinely to patient demographic or clinical data. Hence, they cannot be used to calculate age- and sex-specific prescribing rates or to look at prescribing rates for specific conditions (*18*). We obtained data on all GP prescriptions of floxacillin (including co-fluampicil) and fusidic acid (excluding Fucidin eyedrops but including combined steroid and fucidic acid preparations) for England from 1991 through 2006. We focused on prescriptions of floxacillin and fusidic acid because staphylococcal infection is the only indication given for these conditions in the British National Formulary. We used national population data to calculate crude annual prescribing rates. Analyses used anonymous aggregate data and did not require ethical approval.

## Results

**Figure 1:**
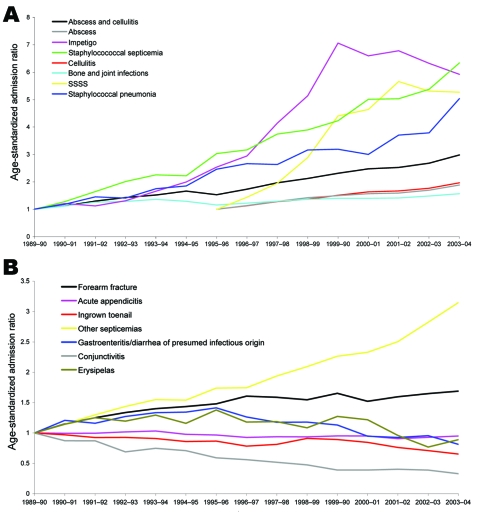
A) Age-standardized admission ratios for community-onset infections identified as or likely to be caused by staphylococci. B) Age-standardized admission ratios for community-onset control conditions. SSSS, staphylococcal scalded-skin syndrome.

For all staphylococcal diseases from 1990–2001 to 2003–04, we found increasing admission trends. These trends are illustrated in Figure 1, panel A, which shows age-standardized hospital admission ratios for suspected staphylococcal disease, and in Table 1, which compares the number of admissions and standardized admission rates in baseline periods and in 2003–04 and shows the standardized admission ratios. For staphylococcal septicemia, staphylococcal pneumonia, impetigo, and SSSS, increases in admission rates were >5-fold over the study period; for abscesses, furuncles, carbuncles, and cellulitis, the increases were nearly 3-fold; for bone and joint infections, the increase was >50%. There were no similar increases in admission rates for the control conditions in general, although there was a 3-fold increase in admissions for septicemia not attributed to staphylococci (Table 1; Figure 1, **panel B**). However, most septicemias recorded in HES have no causative organism specified (68% in 1989–90 vs. 65% in 2003–04), and many of these will in fact have been caused by staphylococci. This lack of data on the organisms that cause septicemia may have masked an even greater increase in staphylococcal septicemias.

For staphylococcal septicemia, staphylococcal pneumonia, abscess, furuncles and carbuncles, cellulitis, and bone and joint infections, both the admission rates and the extent of their rise increased with patient age. Among those >85 years of age, there was a 15-fold increase in admission rates for staphylococcal septicemia, an 8-fold increase in staphylococcal pneumonia, a 4-fold increase in abscesses and cellulitis, and a 2.7-fold increase in bone and joint infections. Nevertheless, major statistically significant increases in admissions for community-onset staphylococcal septicemia, abscess, furuncles and carbuncles, and cellulitis were seen for all age groups. For staphylococcal pneumonia, statistically significant increases were seen for those >16 years of age, whereas for bone and joint infections, they were seen in all age groups except 5- to 15-year-olds. Predictably, impetigo and SSSS were seen primarily in those <16 years of age, and statistically significant increases were confined to children (Table 2).

**Table 2:** Age-specific hospital admission rates (2003–04, per 100,000) and rate ratios (2003–04 vs. baseline) for invasive community-onset staphylococcal infections, England*

Infection	Age group, y							
	All	<4	5–14	15–44	45–64	65–74	75–84	>85
Baseline data 1989–90								
Abscess/cellulitis								
Admission rate	149.3	110.1	45.1	123.2	149.5	216.1	349.5	634.3
Rate ratio (95% CI)	3.0 (2.9–3.0)	1.8 (1.7–1.9)	1.8 (1.7–1.9)	2.7 (2.6–2.8)	3.3 (3.2–3.4)	3.8 (3.6–3.9)	3.5 (3.4–3.7)	4.0 (3.8–4.3)
Staphylococcal septicemia								
Admission rate	3.4	2.2	0.7	1.3	2.7	7.4	14.1	28.7
Rate ratio (95% CI)	6.5 (5.7–7.4)	3.8 (2.2–6.3)	5.5 (2.5–12.2)	6.4 (4.7–8.9)	4.6 (3.5–6.1)	5.4 (4.1–7.2)	7.7 (5.7–10.4)	15.2 (8.9–25.9)
Staphylococcal pneumonia								
Admission rate	1.1	0.7	0.2	0.4	0.7	2.9	5.5	10.0
Rate ratio (95% CI)	5.0 (4.1–6–1)	1.2 (0.6–2.3)	1.3 (0.5–3.2)	4.2 (2.5–6.9)	4.2 (2.5–7.0)	6.0 (3.8–9.7)	9.0 (5.4–15.1)	8.2 (4.2–16.3)
Impetigo								
Admission rate	2.2	24.9	4.2	0.5	0.2	0.1	0.3	0.4
Rate ratio (95% CI)	5.3 (4.6–6.2)	6.1 (5.0–7.3)	11.8 (7.6–18.4)	3.6 (2.3–5.4)	2.1 (0.9–4.7)	1.3 (0.3–4.7)	1.3 (0.4–4.1)	3.2 (0.4–28.2)
Bone and joint infections								
Admission rate	13.4	18.3	9.2	8.5	14.0	22.8	28.6	40.5
Rate ratio (95% CI)	1.6 (1.5–1.6)	1.2 (1.1–1.4)	1.0 (0.9–1.2)	1.4 (1.3 –1.5)	1.7 (1.5–1.8)	2.0 (1.8–2.3)	1.9 (1.7–2.2)	2.7 (2.2–3.4)
Baseline data 1995–96								
Staphylococcal scalded skin syndrome								
Admission rate	1.5	21.3	2.0	0.03	0.03	0.1	0.1	0.1
Rate ratio (95% CI)	4.9 (4.1–5.8)	6.4 (5.2–7.9)	3.7 (2.5–5.4)	1.7 ( 0.5–5.8)	1.9 (0.3–10.2)	3.0 (0.3–29.3)	1.8 (0.2–20.4)	0.2 (0.0–1.6)
Cellulitis								
Admission rate	100.3	50.5	22.9	57.4	108.4	181.6	316.5	595.5
Rate ratio (95% CI)	2.0 (2.0–2.0)	2.2 (2.0–2.4)	1.5 (1.4–1.7)	2.0 (1.9–2.0)	2.0 (1.9–2.1)	2.0 (1.9–2.0)	2.0 (1.9–2.1)	2.0 (1.9–2.1)
Abscess								
Admission rate	491.0	59.6	22.2	65.8	41.2	34.5	33.0	38.8
Rate ratio (95% CI)	1.9 (1.8–1.9)	1.6 (1.5–1.7)	1.5 (1.4–1.7)	2.1 (2.0–2.1)	1.8 (1.7–1.9)	1.7 (1.5–1.8)	1.7 (1.5–1.8)	1.5 (1.3–1.8)

* Years are financial years, April 1 to March 31. CI, confidence interval.

**Figure 2:**
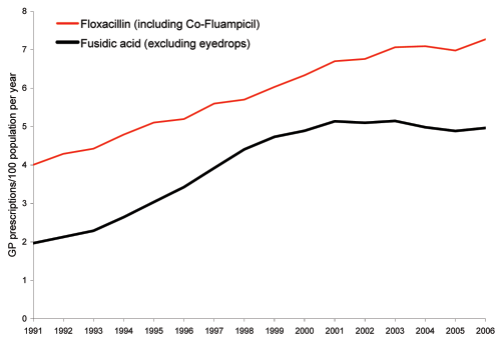
Crude general practitioner (GP) prescription rates (per 100 population), England, 1991–2006.

On the basis of PACT data, a major increase in prescribing for staphylococcal disease by general practitioners was evident (Figure 2). The floxacillin prescribing rate per 100 population was 4.0 prescriptions in 1991 and 7.3 in 2006 (a 1.8-fold increase). The fusidic acid prescription rate per 100 persons was 2.0 in 1991 and 5.0 in 2006 (a 2.5-fold increase).

## Discussion

We have identified a major and previously undocumented increase in community-onset staphylococcal disease. This increase has been ongoing for at least 15 years, since collection of HES data began in 1989, preceding the major (and largely separate) nosocomial increase in MRSA by several years (*19*). The increase has included a wide variety of staphylococcal diseases, has affected all age groups, included both GP prescribing and hospital admissions, and was not matched by similar increases in admission rates for control conditions. There were increasing trends in admissions for septicemia in general, but this increase may have been influenced by the rising incidence of staphylococcal septicemia. Although the increase in admissions has been marked, community-onset staphylococcal infections severe enough to merit admission remain comparatively rare. GP prescriptions for staphylococcal infections have increased to a lesser extent than admissions, but such prescriptions are very common.

Although we cannot be certain that *S. aureus* was the causative organism for all admissions identified here in the HES data, the degree of certainty for those recorded as staphylococcal septicemia, staphylococcal pneumonia, and SSSS is high. *S. aureus* is also the most likely etiologic agent for abscesses, furuncles, and carbuncles (*3*), and bone and joint infections (*6*) and is a very common cause of cellulitis (although β-hemolytic streptococci predominate) (*5*). *S. aureus* is the sole cause of bullous impetigo and the main agent of nonbullous impetigo, where, however, *Streptococcus pyogenes* is sometimes isolated (*20*). In moderate climates such as in the United Kingdom, staphylococcal impetigo is more common, whereas the streptococcal form predominates in warmer and more humid climates (*21*).

National data on voluntary reporting of *S. aureus* bacteremia show a 2.5-fold increase from 1990 to 2004 (an equivalent period to our study) (*22*). This included a 2.1-fold increase in reports of methicillin-sensitive *S. aureus* (MSSA). Our hospitalization data show a much more marked increase in community-onset staphylococcal bacteremia. However, the voluntary reporting system could not distinguish between hospital- and community-acquired bacteremias during this period and therefore cannot give insight into trends in community-onset infections. This means that national surveillance of bacteremia could easily have missed a major increase in community-onset staphylococcal bacteremia. A study from an Oxford hospital laboratory with an estimated referral population of 600,000 identified 697 cases of *S. aureus* bacteremia when the sample was taken within 48 h of admission between 1997 and 2003 but found no evidence of an increase in the numbers of these cases during the study period (*23*). Other research has shown that ≈50% of *S. aureus* bacteremia cases identified at the time of admission are not associated with clinical evidence of septicemia and are thus likely to result from contaminants (*24*). By contrast, our study includes >13,000 admissions in which staphylococcal septicemia was identified as the main clinical reason for the admission and identifies a major increase in these admissions over a longer period. Our study also covers a very much larger population (all of England, ≈50 million persons). The trends in community-onset staphylococcal bacteremia are, in fact, only a small part of a wider trend of increasing hospitalizations for other more common staphylococcal infections and a marked increase in community prescribing for skin and soft tissue infections. Laboratory surveillance, however, tells us very little about trends in these conditions because most are not laboratory confirmed.

Our hospital admission data relate to the primary reason for admission, and the trends identified are therefore for community-onset invasive disease rather than disease arising during periods of hospitalization. This does not necessarily mean that infection was community acquired because infection may only become manifest after the patient is discharged into the community (*15*). We were unable to assess whether the present diagnoses related to previous episodes of hospitalization. However, *S. aureus* is a common cause of infection, and it is unlikely that most of this increase relates to healthcare activity. Previous research has shown that most patients with community-onset MRSA and MSSA bacteremia had a previous history of hospitalization. (However, for MSSA, the average time since last discharge was >100 days, so most patients probably acquired the infection in the community [*23*]). Similarly, trends in community-onset osteomyelitis or abscesses may relate to hospital acquisition, for example, as complications of surgery, but most are not likely to be nosocomial. The trends in admissions for furuncles and carbuncles, cellulitis, impetigo, SSSS, and staphylococcal pneumonia are more likely to reflect real increases in community-acquired infections. Changes in the likelihood of specifying an organism for septicemia are not a probable reason for the increase in staphylococcal septicemia because the proportion of septicemias with a recorded organism (68% in 1989–90 and 65% in 2003–04) did not increase significantly. Ascertainment bias resulting from increased awareness of MRSA may conceivably have led to increased recording of staphylococcal septicemia but is unlikely to have affected recording of community-onset abscesses and boils, cellulitis, impetigo, SSSS, or osteomyelitis because the doctors attending patients and the clerks who code the reasons for admission would be unlikely to associate these with MRSA. Similarly, increased awareness of MRSA cannot account for increases in GP prescribing of floxacillin and fusidic acid.

Our analysis showed a marked increase in admissions of adults for staphylococcal pneumonia. This condition has been linked to the production of PVL toxin (*25*), although nearly 90% of staphylococcal pneumonia cases are not associated with PVL production (*26*). Several outbreaks of PVL-associated infection in the United Kingdom have been well-publicized recently (*27*), but PVL-producing strains of staphylococci are rarely identified in samples other than those from skin and soft tissue infections (*26*). Most skin and soft tissue infections, however, are not microbiologically investigated, and in those for which staphylococci are identified, tests for PVL production are not usually conducted. Testing occurs at the national staphylococcal reference laboratory after referral of isolates. However, referral is usually based on suspicion about the isolate or made because of a local outbreak. Thus, surveillance can miss important increases in the occurrence of PVL-associated disease.

Analysis of routinely collected data on hospital admissions and GP prescriptions has given a unique insight into the macroepidemiology of community-onset staphylococcal disease, impossible through any other means. The large numbers of cases exclude chance as an explanation for the trends. Moreover, the data are representative of England because HES data cover all admissions in the country and PACT data cover all NHS GP prescriptions that are dispensed. Although changes from ICD-9 to ICD-10 complicated the analysis, the underlying trends in pathogenic staphylococcal infections remain clear, and the fact that no equivalent increases in admission rates for unrelated acute community-onset conditions occurred suggests that the increases in staphylococcal disease represent a real shift. The system for collecting national data on hospitalizations and GP prescriptions has remained essentially unchanged throughout the study period. The fact that all NHS hospitals in the country and all community pharmacists and dispensing practices are obligated to use the systems means that the denominator can be accurately determined from census data, which allow measurement of rates. By contrast, in the national voluntary bacteremia reporting system, the size of the denominator is unknown and varies from year to year, depending on which laboratories participate. There are likely to be inconsistencies between hospitals in how diagnostic codes are applied in HES data and how conditions are chosen as the main reason for admission. The absence of major changes in the data collection method throughout the study period, however, means that the large increase in admissions for staphylococcal infections is highly unlikely to be an artifact.

Floxacillin has been the treatment of choice for suspected staphylococcal infections in general practice since the 1970s, so a switch in prescribing from penicillin is not a credible explanation for increasing floxacillin-prescribing rates (*12*). We also note that the increases are in marked contrast to previously reported declines in consultation and antimicrobial prescribing rates for respiratory tract infection over this period (*28*). The increase in GP prescribing mirrors, but is greater than, a similar increase in *S. aureus–*associated skin and soft tissues infections in outpatients in the United States; that increase is hypothesized to be caused by the emergence of community-acquired MRSA, but trends in prescribing in ambulatory care were not examined (*29*). Our data show admissions for skin and soft tissue infections have increased more sharply than GP prescribing for these conditions. This finding could reflect an increase in the average severity of infections.

The yearly increases were relatively modest, but the cumulative effect is a major and previously unrecognized shift in the epidemiology of *S. aureus.* This trend may result from altered virulence or transmissibility of *S. aureus* in general or of particular strains; changes in the host that affect vulnerability (e.g., increasing levels of obesity and diabetes or of intravenous drug use) or transmission dynamics (e.g., increasing use of preschool child care); or changes in the environment, such as widespread use of antimicrobial agents or changes in hygiene behavior. However, these explanations are speculative, and our ignorance of the factors driving such a major change is worrisome, particularly in view of the international concerns about the emergence of community-acquired MRSA and serious PVL-related disease.
